# Dual-Biosensor for Five Drugs Detection in Precision Oncology

**DOI:** 10.1007/s12668-026-02506-8

**Published:** 2026-03-19

**Authors:** Francesca Rodino, Myriam Briki, Thierry Buclin, Monia Guidi, Sandro Carrara

**Affiliations:** 1 Bio/CMOS Interfaces Laboratory (BCI), École Polytechnique Fédérale de Lausanne, 2000 Neuchâtel, Switzerland; 2 Service of Clinical Pharmacology, Department of Medicine and Pathology, Lausanne University Hospital and University of Lausanne, 1011 Lausanne, Switzerland; 3 Centre for Research and Innovation in Clinical Pharmaceutical Sciences, Lausanne University Hospital and University of Lausanne, 1011 Lausanne, Switzerland; 4 Institute of Pharmaceutical Sciences of Western Switzerland, University of Geneva, University of Lausanne, 1206 Geneva, Switzerland

**Keywords:** Biosensors, Electrochemical sensing, CYPs, MWCNTs, TDM, Precision oncology

## Abstract

**Abstract:**

The increasing demand for precision medicine, particularly in oncology, requires innovative solutions to address patient-specific inter-individual variability in drug response. Therapeutic drug monitoring (TDM) is crucial for optimizing treatment efficacy and minimizing toxic side effects, enabling precise dosage adjustments tailored to the patient’s individual metabolic profile. Electrochemical biosensors offer a cost-effective, simple, and portable solution with rapid response times, making them ideal for point-of-care applications. In this work, we propose a novel dual-biosensor platform for TDM, designed to simultaneously detect multiple chemotherapeutic agents–cyclophosphamide, ifosfamide, etoposide, methotrexate, and 5-fluorouracil–for precision oncology. Following real clinical treatment scenarios, the system uses only two working electrodes integrated into a single electrochemical sensing platform, significantly reducing complexity and cost. By integrating MWCNTs with cytochrome P450 enzymes (CYP3A4 and CYP2B6), our platform achieves enhanced electron transfer and substrate specificity, enabling sensitive and selective detection of the five chemotherapeutic drugs, individually and in combination, within clinically relevant ranges. Designed for portability and rapid analysis, this dual-biosensor platform enables real-time, cost-effective drug monitoring at the point-of-care, advancing personalized cancer treatment with greater precision and accessibility.

**Graphical abstract:**

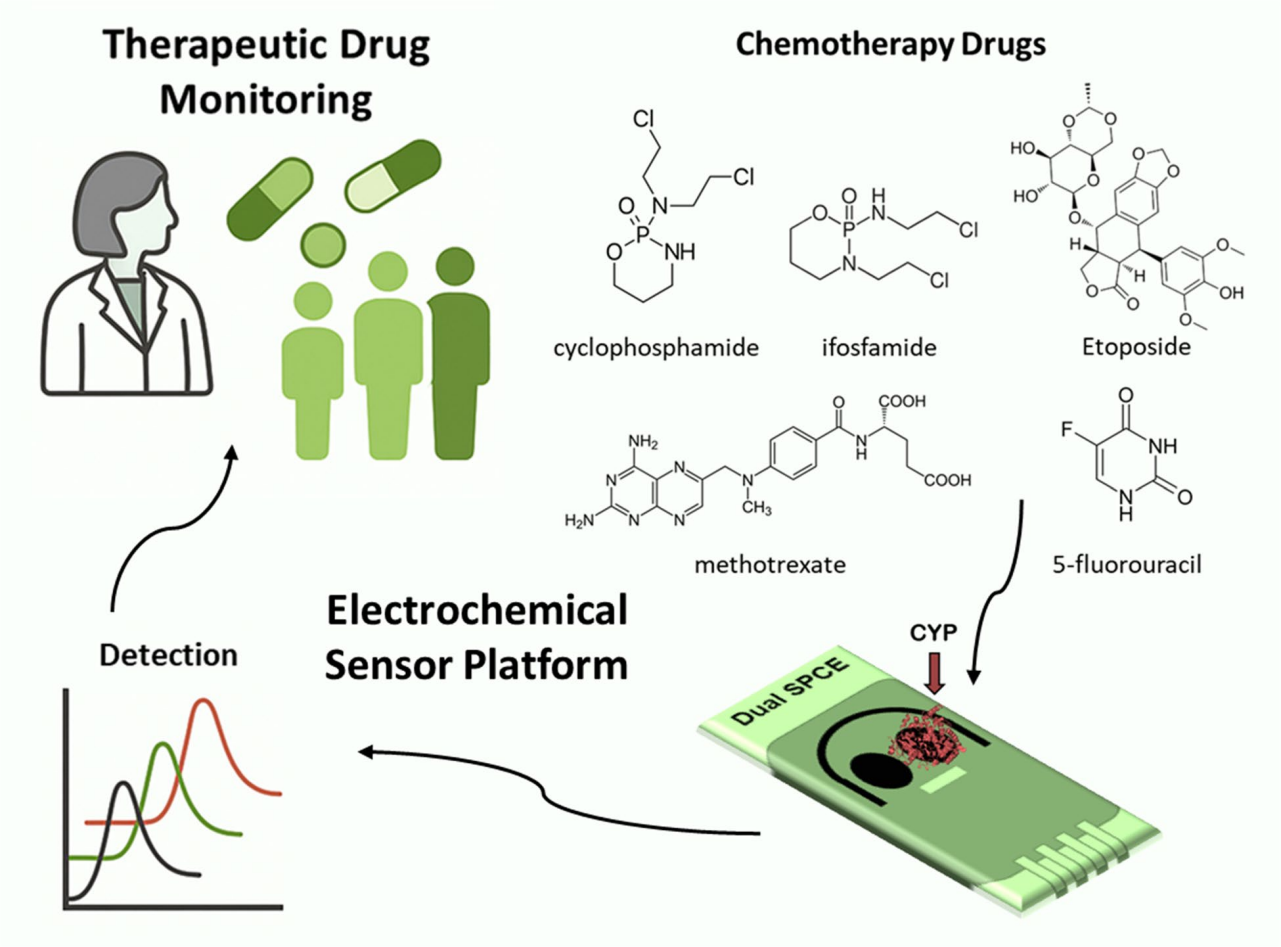

## Introduction

Despite significant advances in drug development and therapeutic strategies, a major challenge remains unsolved in most areas of clinical practice: the high degree of inter-individual variability in drug response [1]. Variations in individual responses to drugs can result from biological, environmental, social, and cultural factors, including variables such as diet, age, sex, body size, concurrent diseases, co-medications, and genetic polymorphisms that affect the pharmacokinetics and/or pharmacodynamics of drugs, leading to different therapeutic outcomes or possible serious side effects. These limitations underscore the pressing need for innovative tools in precision medicine that can dynamically adapt therapies to the patient’s individual pharmacokinetic and pharmacodynamic profile, providing the patient not only with the right drug but also the right dosing schedule to produce the best therapeutic outcome. To this end, therapeutic drug monitoring (TDM) plays a central role, allowing the measurement of drug concentration and the consequent adjustment of dosage [1], reducing toxic side effects, and increasing the effectiveness of treatment [2]. These concepts are particularly important in oncology, where highly powerful and toxic agents are commonly used. Despite the progresses made, chemotherapy remains a fundamental pillar in cancer treatment and one of the most widely used and effective approaches [3]. However, despite the rise of new targeted therapies, their lack of specificity and narrow therapeutic window pose significant challenges for treatment optimization [2, 4]. The therapeutic windows of conventional chemotherapies are often expressed in terms of area under the concentration-time curve (AUC), reflecting overall exposure, rather than peak and/or residual concentrations, more typically used in the monitoring of other treatments. By allowing estimation of the AUC and adapting the dose to fall within targets, TDM of chemotherapeutic drugs can prevent toxicity due to overexposure, not uncommon as the standard dose of most cytotoxic agents corresponds to the maximum tolerated dose [5, 6]. Usually, chemotherapeutic treatments are administered in short cycles over one or a few days, separated by breaks of several weeks, allowing damage to cancer cells to occur while giving healthy cells time to recover. Over one cycle, the monitoring of drug concentrations in the patient’s blood would be beneficial to ensure that the administered doses keep the drug levels within the therapeutic window, between the minimum effective concentration (MEC) and minimum toxic concentration (MTC) and close to the target AUC, thus optimizing treatment efficacy while minimizing the risk of toxicity and harmful side effects [2, 7]. Therefore, the demand for personalized treatments in oncology aligns with precision medicine approaches, where therapeutic decisions are adapted to the individual patient’s characteristics and drug dosing is adjusted accordingly.

The challenge for the development of devices for TDM of chemotherapeutic agents at the point-of-care (PoC) is to provide oncologists with convenient analytical methods ready for use, allowing rapid adjustment of drug dosing before the entire dose is administered. In clinical laboratories, various techniques are used for the analysis of anticancer agents [8]. Separation methods include Liquid Chromatography (LC), Gas Chromatography (GC), Capillary Electrophoresis (CE), and High-Performance Liquid Chromatography (HPLC), while detection methods include ultraviolet spectrophotometry (UV), Mass Spectrometry (MS) and optical methods like surface-enhanced Raman spectroscopy [9] or Surface Plasmon Resonance [10]. While these methods offer reliable data with high reproducibility, they come with significant drawbacks, including expensive instrumentation, high maintenance costs, complex setup, bulky equipment, and long analysis times [11]. These limitations are in contrast with the requirements for PoC technologies to provide rapid measurement and response times in clinics. On the other hand, electrochemical (bio)sensors combined with screen-printed technology offer significant advantages, including cost-efficiency, rapid response, and portability [12], while maintaining good sensitivity and selectivity. Their application to the TDM of chemotherapeutic agents is facilitated by the relatively high doses usually administered, producing substantial circulating concentrations. These qualities make them ideal for drug monitoring and PoC applications [13]. In this context, enzymatic detection offers increased selectivity with fast reaction times. Enzymes are commonly used as recognition elements in electrochemical biosensors due to their high substrate selectivity and sensitivity [14]. Cytochromes P450 (CYPs) stand out for their unique characteristics, making them perfect candidates for enzyme-based detection in amperometric biosensors, especially in the case of drug detection. The catalytic activity of all CYPs is provided by the presence of an inner iron-core, the heme-iron group, in their active site. The heme moiety has a highly specialized lattice structure that supports an iron molecule, which is the core of the enzyme and is responsible for substrate oxidation [15, 16]. CYPs belong to the superfamily of heme-thiolate monooxygenases and play a crucial role in the metabolism of mammals for a wide range of substances, including the detoxification of exogenous bioactive compounds (e.g., carcinogens, drugs, environmental pollutants, food supplements, medicines, etc.) and biotransformation of endogenous bioactive compounds (e.g., amino acids, cholesterol, saturated/unsaturated fatty acids, steroid hormones, etc.) [14, 17]. Although the use of CYPs is proven to add selectivity in drug detection, the enzyme alone is often insufficient to achieve optimal detection sensitivity [18]. To improve their sensitivity, a widely used solution is the integration of nanostructures [13, 16], without compromising the enzyme’s biocatalytic function [19]. In this context, carbon nanotubes (CNTs), particularly multi-walled carbon nanotubes (MWCNTs), stand out for their exceptional electrical properties, making them highly performing nanomaterials to enhance electron transfer in biosensing applications [20, 21]. In biosensing, CNTs offer several benefits, such as small size, larger surface area, high conductivity, chemical stability, high sensitivity, electrocatalytic effect, and fast electron-transfer rate [21, 22]. Not limited to the increase in electroactive surface area, they offer several improved aspects, including a more effective interaction between the target molecules and the sensor’s electrodes [23]. Therefore, by integrating MWCNTs with specific CYP isoforms, the final biosensor benefits from both the enzyme’s substrate selectivity and the nanotubes’ ability to enhance electron transfer with increased sensitivity and maintained enzyme stability. This combination makes MWCNT-CYP biosensors highly effective for detecting drugs and other compounds that require precision monitoring at low concentrations.

In this work, we present a new design approach for a point-of-care biosensing platform capable of monitoring five widely used and established chemotherapy drugs: cyclophosphamide (CP), etoposide (ETO), ifosfamide (IFO), methotrexate (MTX), and 5-fluorouracil (5FU). Unlike conventional systems that rely on complex multi-electrode configurations or single-drug detection, our approach utilizes a dual-electrode configuration to enable the simultaneous and selective detection of multiple chemotherapy agents depending on the specific treatment administered. The proposed biosensors integrate MWCNTs with CYP enzymes–specifically the CYP3A4 and CYP2B6 isoforms, which are involved in the metabolism of IFO and CP, offering high sensitivity and substrate selectivity, ensuring reliable quantification within clinically relevant ranges. Most importantly, we demonstrate that this dual-biosensor platform is capable of addressing treatment regimens typically provided in clinical settings, including drug combinations commonly administered in cancer therapy, thereby paving the way for simplified, cost-effective, and personalized therapeutic drug monitoring at the point of care.

## Materials and Methods

The sensors used are commercially available screen-printed carbon electrodes (SPCEs), model DRP-11L purchased from DropSens, Spain. They consist of a carbon-paste working electrode (WE) (circular shape, 4 mm diameter), a carbon-paste counter electrode (CE), and an Ag/AgCl reference electrode (RE). For the dual-channel measurements, commercial dual-screen-printed carbon electrodes (dual-SPCEs, model DRP-X1110) were also purchased from DropSens. These comprise two separate working carbon electrodes (WE1 and WE2) on the same substrate, sharing a common carbon CE and Ag/AgCl RE. Carboxyl-functionalized multi-walled carbon nanotubes (COOH-MWCNTs) powder was purchased from DropSens (MWCNT, diameter 10 nm, length 1–2 $\mu$m, with 5% –COOH groups content). They were dispersed in chloroform at a concentration of 1 mg/mL and sonicated for 40 minutes in an ultrasonic bath until a homogeneous monodispersion was obtained. Scanning electron microscopy (SEM) was performed to evaluate the morphology and uniform deposition of the MWCNTs on the WE surface. Two different cytochrome P450 isoforms (CYP3A4 and CYP2B6) were purchased from Corning Life Sciences^®^, as isozyme microsomes with CYP reductase and cytochrome b5, recombinant, expressed in baculovirus-infected insect cells (BTI-TN-5B1-4) with a CYP content of 0.5nmol. The microsomes were delivered in a solution containing 100 mM potassium phosphate at pH 7.4 and used without modifications. All the anticancer drugs, cyclophosphamide, ifosfamide, etoposide, 5-fluorouracil, and methotrexate, were purchased in powder form from Sigma-Aldrich^®^. Stock solutions of cyclophosphamide, ifosfamide, and 5-fluorouracil were prepared in deionized water, while etoposide and methotrexate were dissolved in dimethylsulfoxide due to their low solubility in water. Samples containing single drugs were prepared in different concentrations in 0.1 M phosphate-buffered saline (PBS), while samples containing drug combinations were prepared in 0.1 M phosphate buffer (PB). NaOH (0.1M) was added to slightly adjust the pH to 7.4, emulating physiological conditions and preserving the enzymatic activity and integrity of the CYPs. All chemicals were purchased from Sigma-Aldrich, unless otherwise specified. Electrochemical measurements were performed using the Autolab PGSTAT101 (Metrohm, Switzerland) controlled by Nova 2.1 software. Cyclic voltammograms were acquired under aerobic conditions after drop-casting 100 $\mu$L of the testing sample on the SPCE. The applied potential was swept in the range from -0.6 V to 1 V, with a scan rate of 20 mV/s. Cyclic voltammetry (CV) and Differential Pulse Voltammetry (DPV) techniques were employed for the electrochemical measurements due to their ability to simultaneously and selectively identify multiple substances through their well-distinct redox peaks. To determine the current peaks, baseline subtraction was applied using asymmetric least-squares smoothing and a spline interpolation curve in the Origin Pro software. Sensitivity (S) and limits of detection (LoDs) are derived from the calibration curves; in particular, LoD was calculated as $3*\sigma _{blank}/S$, where $\sigma _{blank}$ represents the standard deviation of the blank solution and *S* is the slope of the calibration curve obtained. Reproducibility was assessed using three independently electrodes (n=3) for each condition; reported values (and error bars where shown) represent the mean and standard deviation across electrodes.

### Preparation of MWCNT/CYP Electrodes

The biosensors were functionalized by drop-casting deposition of 30$\mu$L of a 1 mg/mL MWCNT suspension onto the WE. This process was performed gradually by depositing single 5$\mu$L drops to control the rapid spreading of the chloroform on the electrode’s surface. After each deposition, the chloroform easily evaporates, leaving only the nanotubes on the WE surface, forming a 3D porous nanostructure. After the MWCNTs deposition, CYP3A4 or CYP2B6 was drop-casted onto the nanotubes’ matrix. A total of 9 $\mu$L of CYP solution was deposited in three steps of 3 $\mu$L each. After each deposition, the electrodes were stored in the fridge at 4$^{\circ }$C overnight to ensure complete adsorption of the enzyme. The successful modification of the electrodes was subsequently evaluated through SEM imaging and electrochemical characterization, as described in detail in the Results and Discussion section.

## Results and Discussion

### Surface Characterization of MWCNT Electrodes

**Fig. 1:**
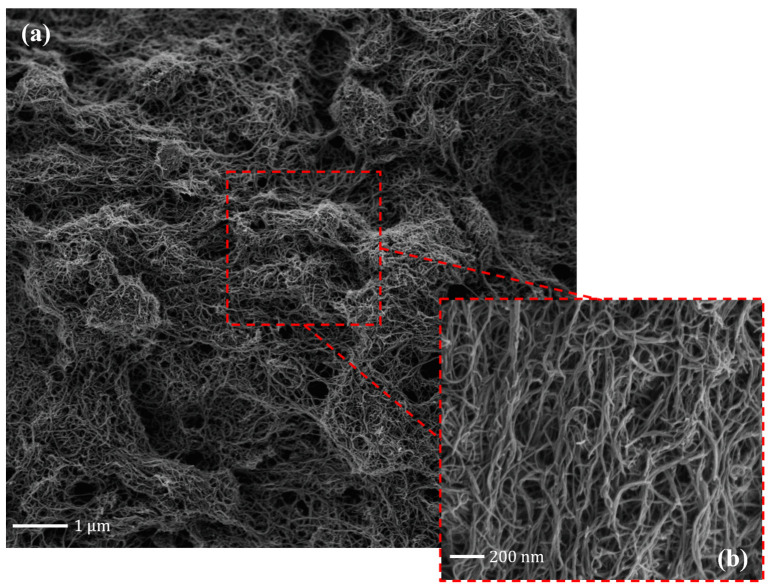
SEM images of the MWCNT distribution on the working electrode surface. Images were acquired at 20,000$\times$ (**a**) and 100,000$\times$ (**b**) magnification. Scale bars are shown in each panel

Figure 1 presents SEM images of the carbon WE surface after the deposition of the MWCNTs. Scanning electron microscopy (SEM) images were acquired using a Gemini 2 field-emission scanning electron microscope (ZEISS, Germany). Imaging was performed at an accelerating voltage (EHT) of 1 kV, with a probe current of 60 pA, using the InLens detector and a working distance of 2.5 mm. Images were collected at magnifications of 20,000$\times$ (Fig. 1a) and 100,000$\times$ (Fig. 1b), revealing a uniform distribution of MWCNTs across the electrode surface. The higher-magnification image confirms that the nanotubes are well-dispersed and without residual aggregates.

**Fig. 2:**
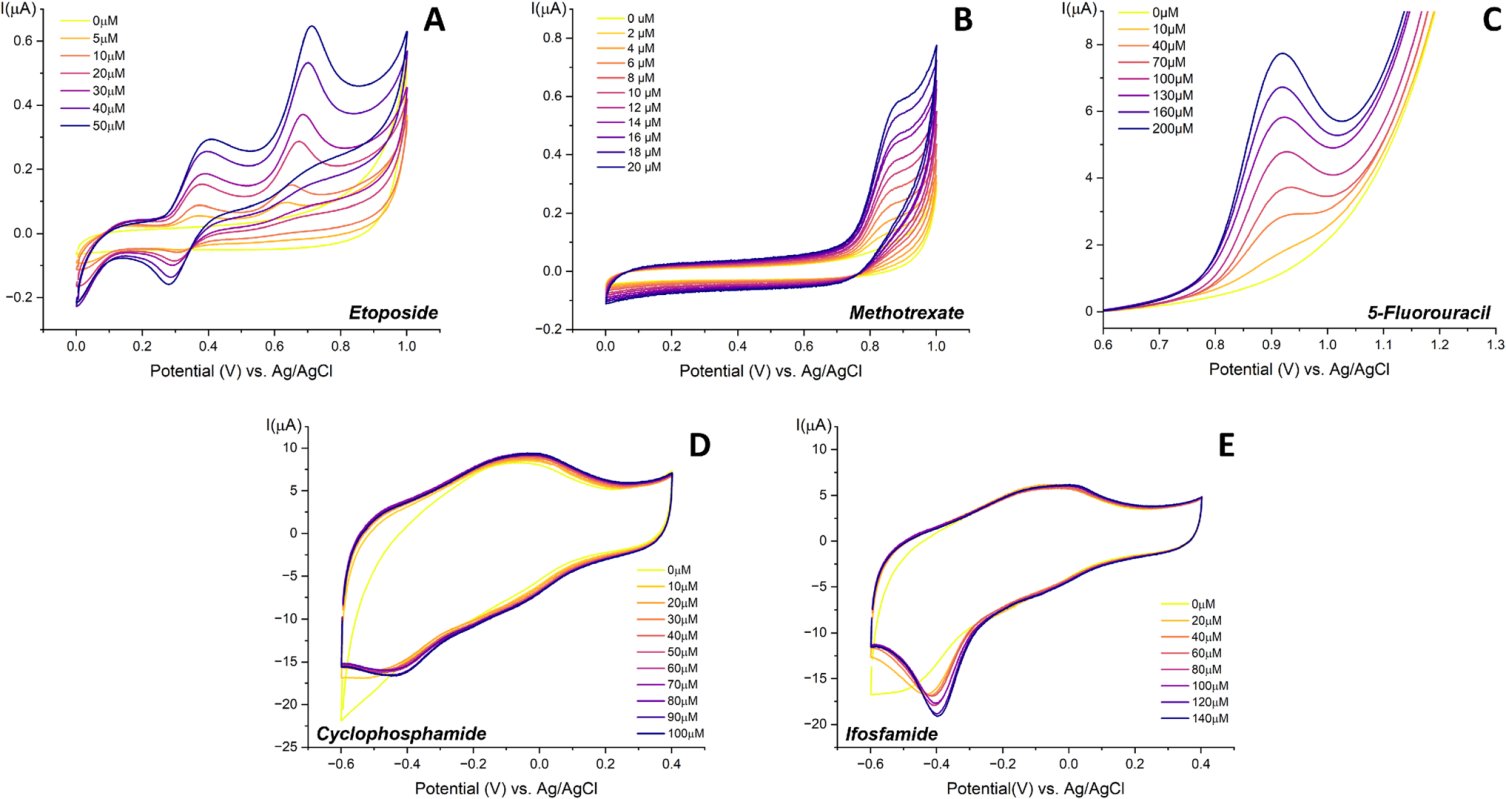
Voltammograms of the five anticancer drugs measured within their clinically relevant concentration ranges under aerobic conditions at the scan rate of 20 mV/s. Etoposide (**A**), Methotrexate (**B**), 5-Fluorouracil (DPV) (**C**), MWCNT/CYP2B6 electrode for cyclophosphamide (**D**), MWCNT/CYP3A4 electrode for ifosfamide (**E**)

### Single Drugs Detection

In order to validate the proposed multi-drug platform, each chemotherapeutic agent was first investigated individually within its clinically relevant concentration range. Two distinct detection mechanisms were explored: direct electrochemical oxidation for intrinsically electroactive drugs (ETO, MTX, 5FU), and CYP-mediated catalytic detection for prodrugs requiring metabolic activation (CP, IFO). This step establishes the analytical foundation necessary before addressing multi-drug combinations.

#### Non-Enzymatic Detection (ETO, MTX, 5FU)

We first evaluated drugs that exhibit intrinsic electrochemical activity and can therefore be directly detected on bare carbon electrodes, without requiring enzymatic functionalization. In these cases, the analytical signal arises from direct oxidation of the drug.

##### Etoposide

Etoposide belongs to the class of topoisomerase II inhibitors, which exert their cytotoxic action by interfering with the activity of topoisomerase enzymes, crucial for DNA replication and repair processes [11]. By inhibiting this enzyme, often more active in rapidly dividing cells, etoposide induces DNA damage, selectively affecting malignant cells. This selective toxicity makes etoposide particularly useful in malignancies where topoisomerase II activity is significantly upregulated, such as certain types of lung and testicular tumors, as well as leukemias and lymphomas. Based on pharmacological studies, the measures of ETO in patients’ plasma are expected to be observed between 30$\mu$M and 130$\mu$M approximately [24, 25]. Additionally, the study conducted by Tazawa et al. reports minimum measured concentration levels with median values ranging from 6.2 $\mu$M to 9.2 $\mu$M [25]. According to the literature, ETO metabolism is primarily mediated by CYP3A4 [26]. However, ETO is also an electroactive drug, meaning that it is prone to exchange electrons without the intervention of enzymes. Therefore, a non-enzymatic sensing approach was chosen for two reasons: to avoid substrate competition between two drugs on the same enzyme, and to simplify the system.

Figure 2A shows the CV of ETO on a bare carbon electrode at increasing concentrations from 5 $\mu$M up to 50 $\mu$M. For this study, a smaller concentration detection range was preferred to verify sensor performance at lower concentrations than those previously reported in the literature [13], with the aim of preventing and correcting cases of ineffective dosing.

**Fig. 3:**
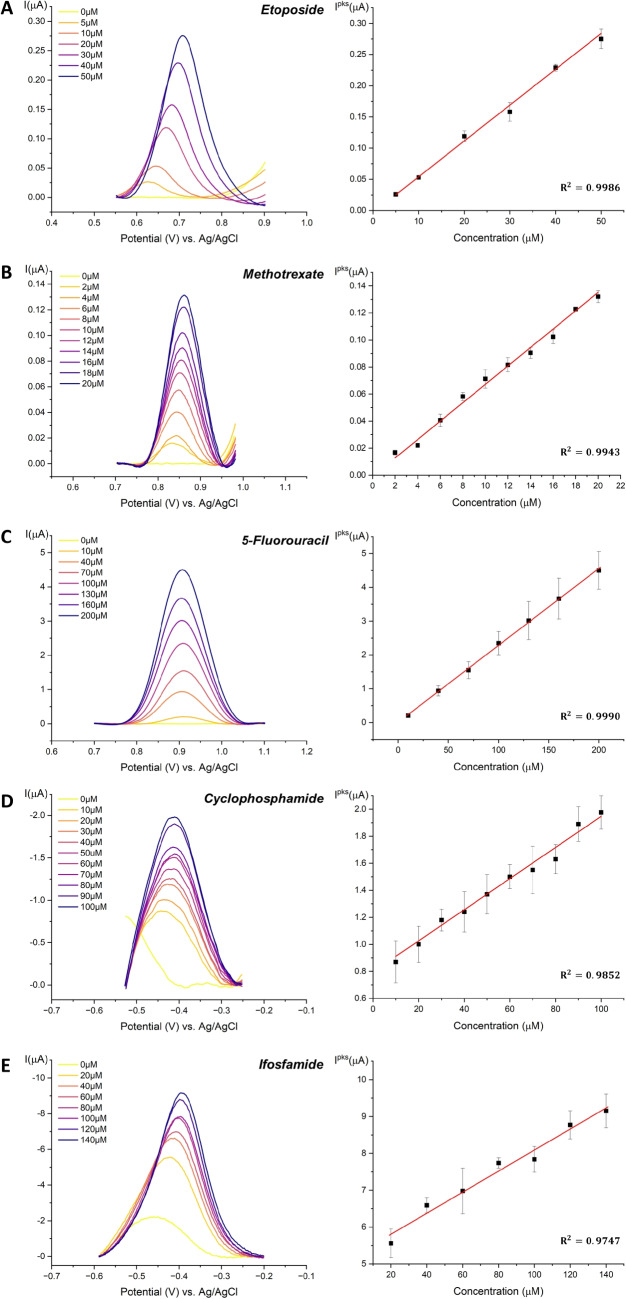
Baseline-subtracted peaks (left) and calibration curves (right), obtained from voltammetric measurements in PBS with increasing drug concentrations. Error bars represent the standard deviation across three prepared electrodes (n=3)

The oxidation of ETO on the SPCE results in two oxidation peaks at 400 mV and 700 mV and a reduction peak at 280 mV, which is consistent with previous findings reported in the literature [27]. Figure 3A shows the second anodic peak at 700 mV after baseline subtraction. This peak was preferred due to its higher current response with respect to the peak at lower potentials. Figure 3A (right) presents the corresponding calibration curve resulting in a sensitivity equal to $S=5.75\,nA/\mu M$, with excellent linearity $R^2=0.9986$.

##### Methotrexate

Methotrexate belongs to the class of folate antagonists, interfering with the metabolism of folic acid and inhibiting folate-dependent enzymatic activity, ultimately disrupting the production of deoxyribonucleic acids, essential building blocks for cell replication [11]. TDM of MTX concentration is routinely performed when using high doses followed by folinic acid (Leucovorin) rescue. Indeed, MTX serum levels essentially guide the administration of folinic acid and the hydration needed to prevent toxicity, and TDM is performed until MTX concentrations decrease to approximately 1$\mu$M, depending on the protocol [28, 29]. Another crucial aspect is to monitor drug elimination to prevent the associated adverse effects and toxicity risks. For example, patients with serum MTX levels of less than 10, 1, and 0.1 $\mu$mol/L at 24, 48, and 72 hours after infusion, respectively, are considered to have normal elimination, whereas higher levels indicate delayed elimination and increased risk of toxicity [28].

Clinically observed ranges are reported between sub-$\mu$M concentrations up to more than $10^{3} \mu$M [30]. However, there are specific sub-ranges of particular interest, as highlighted by [28], where MTX monitoring is crucial for the coadministration of Leucovorin. Specifically, in treatments for acute lymphoblastic leukemia, Leucovorin doses were adjusted 42 hours after high-dose MTX infusion based on MTX concentrations and patient weight, especially when levels exceeded 5 $\mu$mol/L. In addition, in patients with acute lymphoblastic leukemia who achieved a steady-state plasma concentration of MTX above 16 $\mu$mol/L, complete remission rates significantly increased, and relapse rates decreased [30]. In the same study, it has been reported that TDM remains of great interest until MTX concentration falls below 1 $\mu$mol/L. Based on these findings, we selected a detection range of 2-20 $\mu$M as it covers key MTX concentrations critical for guiding Leucovorin dosing and managing treatment during the post-infusion period.

Similarly to the case of ETO, MTX is also naturally electroactive, thus detectable without the help of any enzyme.

Figure 2B shows the CV of MTX in the range of 2 $\mu$M up to 20 $\mu$M, demonstrating that the drug can be well detected even at very low concentrations. From the graph, it is evident that the redox behavior of MTX exhibits one single anodic peak at 850 mV. Figure 3B highlights the increasing peaks after baseline subtraction and its calibration curve. The sensor exhibits very high linearity, with $R^2=0.9943$, and a sensitivity of $S=6.81 nA/\mu M$.

##### 5-Fluorouracil

5-Fluorouracil is a chemotherapeutic agent used in the treatment of a wide variety of malignancies such as gastric adenocarcinoma, pancreatic adenocarcinoma, breast carcinoma, and colorectal adenocarcinoma [31]. Its TDM is widely encouraged, with recommended guidelines that allow the adjustment of the dose of 5FU for the next planned cycle depending on the exposure during the current cycle [32]. While the typical expected concentration range in patients’ plasma can vary from sub$\mu$M (0.1 $\mu$M) up to 75 $\mu$M [33, 34], it is also highlighted the significant fluctuations in plasma levels and high interindividual variability in patients’ responses [34, 35]. For example, Casale et al.[36] report peaks around 400 $\mu$M in colon cancer patients shortly after infusion, with levels rapidly decreasing to approximately 75 $\mu$M and then to a few $\mu$M within the first hours. Therefore, we selected a detection range of 10-200 $\mu$M for the measurement of 5FU to account for inter-patient variability covering a wider range. For the detection of this drug, DPV was performed to emphasize the faradic peak, enabling the detection at a lower concentration that would not have been possible with CV alone. The results are presented in Figs. 2C and 3C, where the peak is highlighted after baseline subtraction with the corresponding calibration curve. The oxidation peak at 930 mV, related to the oxidation of 5FU, aligns with previous observations in the literature [37]. The sensitivity is equal to S = 22.75 $nA/\mu$M with an excellent linear response equal to $R^2=0.9990$.

Together, these results demonstrate reliable and sensitive detection of electroactive chemotherapeutic agents using a non-functionalized SPCE platform.

#### Enzymatic Detection (CP, IFO)

In contrast, cyclophosphamide and ifosfamide do not exhibit a sufficiently direct electrochemical response within the investigated potential window and clinically relevant concentration range. Therefore, enzymatic activation through CYP-functionalized electrodes is necessary to enable sensitive and selective electrochemical detection.

##### Cyclophosphamide

Cyclophosphamide is a chemotherapy drug that belongs to the class of alkylating agents. It is commonly used in the treatment of various malignancies, including leukemia, lymphoma, and breast cancer [38]. As a prodrug, CP requires metabolic activation by hepatic cytochrome P450 enzymes, primarily CYP2B6, resulting in the formation of 4-hydroxycyclophosphamide. This metabolite rapidly equilibrates with its tautomer, aldophosphamide, which then diffuses into cells, where it spontaneously decomposes into the active cytotoxic agent phosphoramide mustard, responsible for DNA alkylation [39–41]. In more detail, approximately 70–80% of the administered CP dose is metabolized to 4-hydroxycyclophosphamide, primarily by CYP2B6, with minor contributions from CYP2C19 and CYP3A4 [13, 41, 42].

Chen et al. [43] describe whole blood concentrations of CP varying between 10 and 400 $\mu$M. Furthermore, plasma concentration-time profiles commonly show an initial peak concentration of 200 $\mu$M, immediately followed by a rapid decrease to around 100 $\mu$M within a few hours, and then a gradual decline over time to values around 50 $\mu$M [44]. Accordingly, we selected a detection range of 10–100 $\mu$M for this study. Figure 2D shows the CV performed with the prepared MWCNT/CYP2B6 in the presence of CP in PBS at increasing concentrations from 10$\mu$M up to 100$\mu$M. Here, the characteristic curve associated with cytochrome P450 activity can be recognized, reflecting its electrocatalytic activity originating from changes in the heme oxidation state of the heme iron nucleus, which is responsible for the direct electron transfer from the heme iron to the electrode surface [14].

In the presence of dissolved oxygen, which acts as a co-substrate of CYP, the voltammogram is characterized by a clear increase in the reduction peak current and a minor oxidation peak near the zero potential (Fig. 2D, E). This is explained by the fact that, under aerobic conditions, the reduced form of CYP (Fe(II)) binds rapidly to oxygen, forming a transient and highly reactive ferrous–dioxygen complex (Fe(II)–$O_{2}$). This complex readily accepts a second electron, leading to its oxidation back to the ferric form (Fe(III)), which can then undergo further reduction, resulting in more pronounced reduction currents. However, because Fe(II) is quickly captured by $O_{2}$, less Fe(II) remains available for direct electrochemical reoxidation. As a result, the oxidation peak corresponding to the Fe(II) $\rightarrow$ Fe(III) transition is still visible but significantly diminished, as observed in the cyclic voltammograms in Fig. 2D and E [16, 45]. When a substrate, or a drug in this case, is introduced into an oxygenated buffer solution, a further increase in CYP reduction current is observed. This increase is proportional to the substrate concentration, enabling the measurement of catalytic current, thus allowing for the detection and quantification of the substrate.

From the figure, it can be observed that in the presence of CP, the reduction peak at -410 mV increases with the increased concentration of the drug. Figure 3D highlights the increasing cathodic peak due to the presence of CP at -410mV after baseline subtraction. This result confirms the correct detection of CP by exploiting the CYP2B6 enzyme, as it is also displayed by the calibration curve in Fig. 3D (right). The biosensor demonstrates a high linearity with a $R^2$ value of 0.9852 and sensitivity equal to $S=11.81\,nA/\mu M$, which aligns with previous literature findings [13].

##### Ifosfamide

Ifosfamide, like cyclophosphamide, is an alkylating agent used to treat malignant diseases such as lymphoma, sarcoma, and lung cancer [46]. Similarly to CP, IFO is a prodrug that requires metabolic activation by hepatic CYP enzymes. Its primary activation pathway involves hydroxylation at the ring’s carbon-4 position, predominantly catalyzed by CYP3A4, with minor contributions from CYP2B6 and CYP2C9. This reaction produces the active metabolite 4-hydroxyifosfamide, which subsequently converts to aldoifosfamide and spontaneously decomposes into the DNA-alkylating agent isophosphoramide mustard, thus inhibiting cancer cell proliferation [47–49].

Although clear therapeutic targets for IFO or its metabolites are not yet available, the variability in its pharmacokinetics supports the use of TDM to detect significant deviations from typical population values. Additionally, data from a reliable and simple analytical method could help establish reference ranges by collecting more information on PK-PD relationships [2]. Reported clinically observed concentration ranges of IFO vary across the literature, depending on the method of drug administration and the dose. For example, according to Singer et al. [50], the maximum concentration can reach approximately 380 $\mu$M for rapid intravenous bolus infusion, compared to around 185 $\mu$M for continuous, slower infusion. Nevertheless, most studies converge in reporting an average of maximum concentration of about 300 $\mu$M [49, 51], with minimum concentrations of a few $\mu$M. Accordingly, a detection range between 10 $\mu$M to 140 $\mu$M was selected for this study to validate the proper functioning of the biosensor in a median concentration range relative to both infusion methodologies with respect to the serum concentration values reported in the literature [50, 51]. Similarly to CP, a specific biosensor based on MWCNT/CYP3A4 was designed for IFO detection. The results are shown in Fig. 2E, which presents the CV performed in the presence of IFO in PBS at increasing concentrations from 20 $\mu$M up to 140 $\mu$M.

As previously discussed for CP, the typical curve produced by the involvement of cytochrome P450 can be recognized, and finally, the cathodic peak at -400 mV increases with increasing drug concentration, confirming its successful quantification. Figure 3E highlights the increasing reduction peak due to the presence of IFO at -400 mV after baseline subtraction and the calibration curve, respectively. The biosensor response yields a very high linear current response with a sensitivity of $S=28.55\,nA/\mu M$ and an $R^2$ value of 0.9747.

The distinct cathodic peaks observed for CP and IFO confirm that CYP-functionalized electrodes enable selective detection through a mechanism operating in a separate electrochemical potential domain from the oxidation-based signals of ETO, MTX, and 5FU. This separation in the electrochemical potentials suggests the possibility of spatially separating the two detection mechanisms within a single detection platform.

**Table 1:** Summary of biosensors’ performances for the selected anticancer drugs compared to reported ranges observed in patients’ plasma

Drugs	CP	IFO	ETO	MTX	5FU
Sensitivity (nA/$\upmu$M)	11.81	28.55	5.75	6.81	22.75
LoD ($\upmu$M)	0.98	5.26	0.71	0.75	2.97
Reported range ($\upmu$M)	10–200	10–300	5–100	0.05–2700	0.1–75
	[43, 44, 52]	[49–51]	[24, 25]	[30]	[33]

Table 1 summarises the sensing performances of the biosensors developed for the detection of the five anticancer drugs CP, IFO, ETO, MTX, and 5FU, including sensitivity, LoD, and comparison with clinically reported concentration ranges. In particular, for CP and IFO, enzymatic functionalization was essential to obtain an analytical response. In the absence of CYP, no quantifiable electrochemical signal was observed on bare SPCEs. In contrast, MWCNT/CYP-modified electrodes enabled measurable catalytic sensitivities of 11.81 nA/$\upmu$M for CP and 28.55 nA/$\upmu$M for IFO (Table 1), highlighting the critical role of enzyme-based nanostructuring for the detection of these drugs. In addition to the high sensitivity, the biosensors also demonstrated reliable short-term stability during measurements, with low standard deviations recorded during calibration, consistent with stable enzyme immobilization on COOH-MWCNTs [16, 53]. Furthermore, for the electroactive drugs, ETO and MTX demonstrated well-defined oxidation peaks with excellent linearity, while DPV-based detection of 5FU provided improved faradaic signal resolution.

The LODs presented in Table 1 confirm the biosensor’s ability to detect all five drugs within their respective clinically relevant concentration ranges. In fact, ETO, MTX, and CP show very low LoDs, making the corresponding biosensor suitable for clinical monitoring applications. For MTX, the LoD of 0.75 $\mu$M supports quantification within the selected clinically relevant range (2–20 $\mu$M), which is critical for dose adjustment and post-infusion monitoring. However, further sensitivity improvements would be required to reliably reach the lowest thresholds used to guide discontinuation of Leucovorin rescue (e.g., 0.05 $\mu$M) in certain clinical protocols [28]. Moreover, even if the biosensor for IFO has a slightly higher LoD with respect to CP, it remains below the lower boundary of the typical therapeutic concentration range. The measurement of 5FU, despite its high sensitivity and maximum linearity, shows a detection limit that, although low, remains above the lower limit of the clinically observed range. To address this limitation, the integration of highly conductive nanomaterials such as the use of platinum (Pt) nanostructures, could enhance sensitivity and response time [54]. Overall, these results demonstrate reliable analytical performance in terms of sensitivity and limits of detection within a range of clinically relevant concentrations for the selected chemotherapeutic drugs.

Finally, it is worth mentioning the fundamental difference in the working potentials associated with the two detection mechanisms used in this study: enzymatic and non-enzymatic. ETO, MTX, and 5FU exhibit intrinsic electrochemical activity and undergo direct oxidation at positive potentials. This is because electron removal from stable molecular orbitals requires an energetically favorable potential, allowing the electrode to act as an electron acceptor and drive the oxidation process. Instead, CYP-based detection relies on the catalytic activity of cytochrome P450 enzymes, which operate through the redox activity of the heme-iron center of CYP450. This process results in the characteristic cathodic peak around -400 mV, corresponding to the reduction of the heme-iron complex within the enzyme, as seen in the detection of CP and IFO.

**Table 2:** Examples of available treatment regimens involving a combination of the studied drugs

Treatment	Drugs combination	Indication	Ref
EPOCH	CP, ETO	Lymphomas	[55, 56]
ICE	ETO, IFO	Lymphomas	[57, 58]
MIED	ETO, IFO, MTX	Recurrent or refractory lymphomas	[59]
CMF	CP, MTX, 5FU	Breast Cancer	[60]

### Multiple Drug Detection

The complexity of chemotherapy treatments lies in the fact that they involve the administration of not just one drug, but a combination of multiple chemotherapy agents. This presents a major challenge for TDM in clinical applications. This requires the simultaneous detection of multiple anticancer agents in order to ensure accurate monitoring and significantly improve patient safety. Commonly administered oncology treatments are exemplified in Table 2. The combination of ETO and MTX is used in MIED therapy, while 5FU and MTX are frequently co-administered with CP in the case of CMF treatment.

**Fig. 4:**
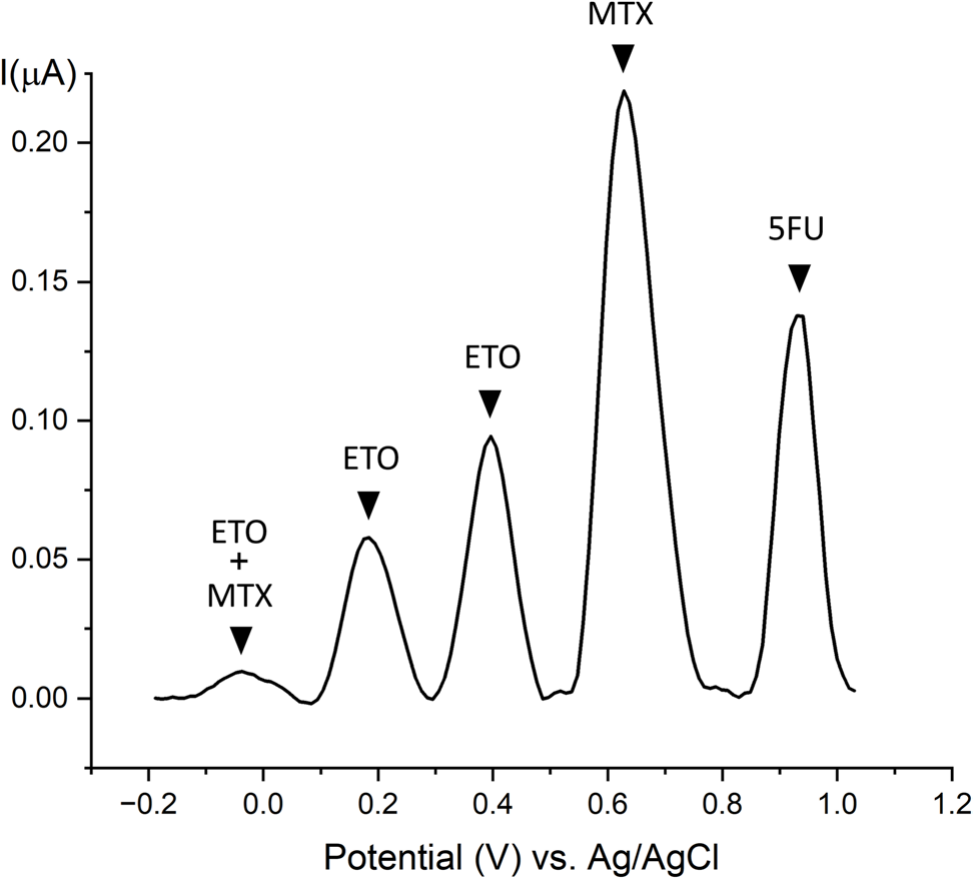
Simultaneous detection of three anticancer drugs: etoposide, methotrexate, and 5-fluorouracil, with one electrochemical sensor. The distinct peaks of ETO, MTX and 5FU remain clearly detectable, allowing their differentiation according to different oxidation potentials

In our previous work [11], we showed and deeply discussed the detection obtained when ETO and MTX are simultaneously present in the same sample. The distinct oxidation peaks of ETO and MTX at approximately 400 mV, 700 mV, and 900 mV, respectively, remained clearly detectable, allowing for their differentiation based on potential. Additionally, a fourth peak at lower potentials (around 150 mV) was attributed to the electrochemical oxidative cross-reactivity of the drugs together within an electrochemical system.

According to Table 2, MTX can be prescribed together with ETO in MIED treatment and together with 5FU in CMF treatment; therefore, we investigated the behavior of these three drugs together present in the same sample on a single sensor. Figure 4 presents the current response of a carbon electrode used for the simultaneous detection of ETO, MTX, and 5FU. The figure depicts the faradic peaks after baseline subtraction. The distinct peaks corresponding to ETO, MTX, and 5FU are clearly identified, indicating successful differentiation of the three compounds. The ETO peaks appear at approximately 200 mV and 400 mV, MTX at around 700 mV, and 5FU at approximately 950 mV. The additional peak observed near 0 V is attributed to mutual electrochemical interference between ETO and MTX, resulting from their oxidative cross-reactivity when simultaneously present [11]. The voltage peak shifts observed during simultaneous detection, compared to individual measurements, are due to the use of a chloride-free phosphate buffer. This buffer was selected to minimize unrelated interferences and provide a more stable environment for studying drug–drug interactions. This shift, while expected, does not affect the sensors’ performance, as the distinct peaks for each drug confirm its effectiveness for multi-drug detection.

The ability to selectively and simultaneously detect ETO, MTX, and 5FU within a single electrochemical channel, combined with CYP-mediated catalytic detection for CP and IFO, naturally suggests the spatial separation of these mechanisms within the same detection platform. This observation forms the basis for the development of the dual-channel architecture proposed below.

#### Dual-Channel Architecture for Multi-Drug Monitoring

**Fig. 5:**
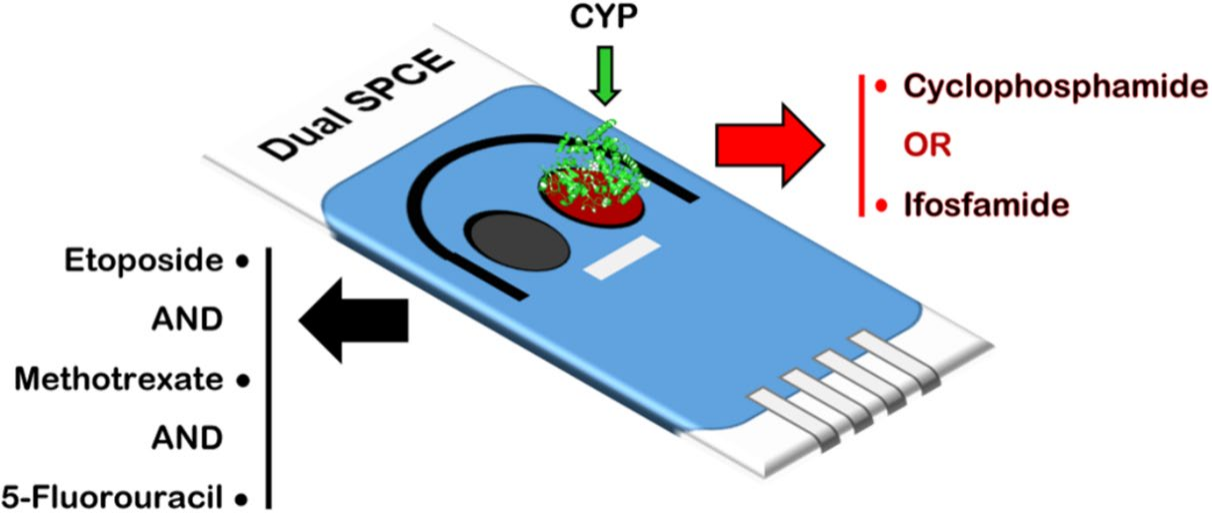
Proposed dual-biosensor for the simultaneous detection of multiple drugs depending on the combination administered, using a single electrochemical sensing platform

Building upon the demonstrated simultaneous detection capability of the non-enzymatic channel and the enzyme-dependent detection mechanism of the CYP-based sensors, we propose a dual-channel architecture for integrated multi-drug monitoring. This configuration enables selective quantification of chemotherapeutic combinations within a single sensing platform, addressing the analytical demands of therapeutic drug monitoring in oncology. In this architecture, three of the five investigated drugs (ETO, MTX, 5FU) are detected via direct oxidation on the non-enzymatic channel, while CP and IFO are quantified through CYP-mediated catalytic detection on the second channel. Figure 5 illustrates the dual-SPCE configuration designed to enable analysis of mixed drug samples within a single measurement, reflecting clinically relevant scenarios. The figure shows a single electrochemical sensor with two active channels: one for non-enzymatic detection (WE1, dark gray electrode) and the second for enzymatic detection using electrodes modified with MWCNTs/CYP (WE2, red electrode). This approach enables the selective recognition and further quantification of multiple drugs in a single sample. For instance, CP or IFO can be detected on WE2, while other drugs, ETO, MTX, and 5FU, can be detected on WE1. With this configuration, it is possible to obtain comprehensive detection of the targeted chemotherapy drugs according to the type of oncological treatment used, using a single electrochemical sensor. One potential concern is whether interference between drugs could occur across the two detection channels. According to our findings, detecting CP and IFO in the required therapeutic range is not feasible without appropriate enzymes functionalization [61], meaning that no significant interference can occur on the non-enzymatic channel. However, an interesting question is whether drugs such as ETO, MTX, or 5FU can compete with CP or IFO on the enzyme-based channel. Based on currently available information, there is no evidence that MTX or 5FU undergo CYP-driven metabolism [62–64], whereas ETO is known to be a substrate for CYP3A4 [13]. Although this may be seen as problematic, the interaction between ETO and IFO can be controlled. In fact, it has already been demonstrated that, while the concentration of IFO is affected by the presence of ETO due to substrate competition, the concentration of ETO is not found to be influenced by the presence of IFO [13]. This means the sensor’s response can be properly calibrated in post-processing to account for potential interference, thus ensuring accurate detection. An additional consideration concerns the simultaneous detection of IFO and CP, as they are both detected via CYP-based enzymatic sensing and produce similar cathodic peaks, which may complicate their distinction during concurrent analysis. However, based on clinical treatment protocols, these two drugs are very rarely co-administered. As shown in Table 2, CP and IFO are typically not used together, except in rare cases such as aggressive or high-risk lymphomas, where multiple treatment regimens like CODOX-M/IVAC may be combined [65]. However, even in such scenarios, CP and IFO are administered in alternating cycles rather than concurrently, allowing the body to clear one drug before the next is introduced [66]. This sequential administration prevents the risk of overlapping plasma concentrations and, therefore, the potential interference during their detection, preserving the specific detection of each drug within its respective treatment cycle. Finally, it is worth noting that cytochrome P450 enzymes catalyze irreversible oxidative transformations of CP and IFO; consequently, the enzymatic detection mechanism is not reversible. For point-of-care clinical applications, this does not represent a limitation, as the electrochemical sensing element is designed as a single-use component to avoid sterilization procedures, minimize cross-contamination, and variability associated with enzyme activity decay during repeated use.

**Fig. 6:**
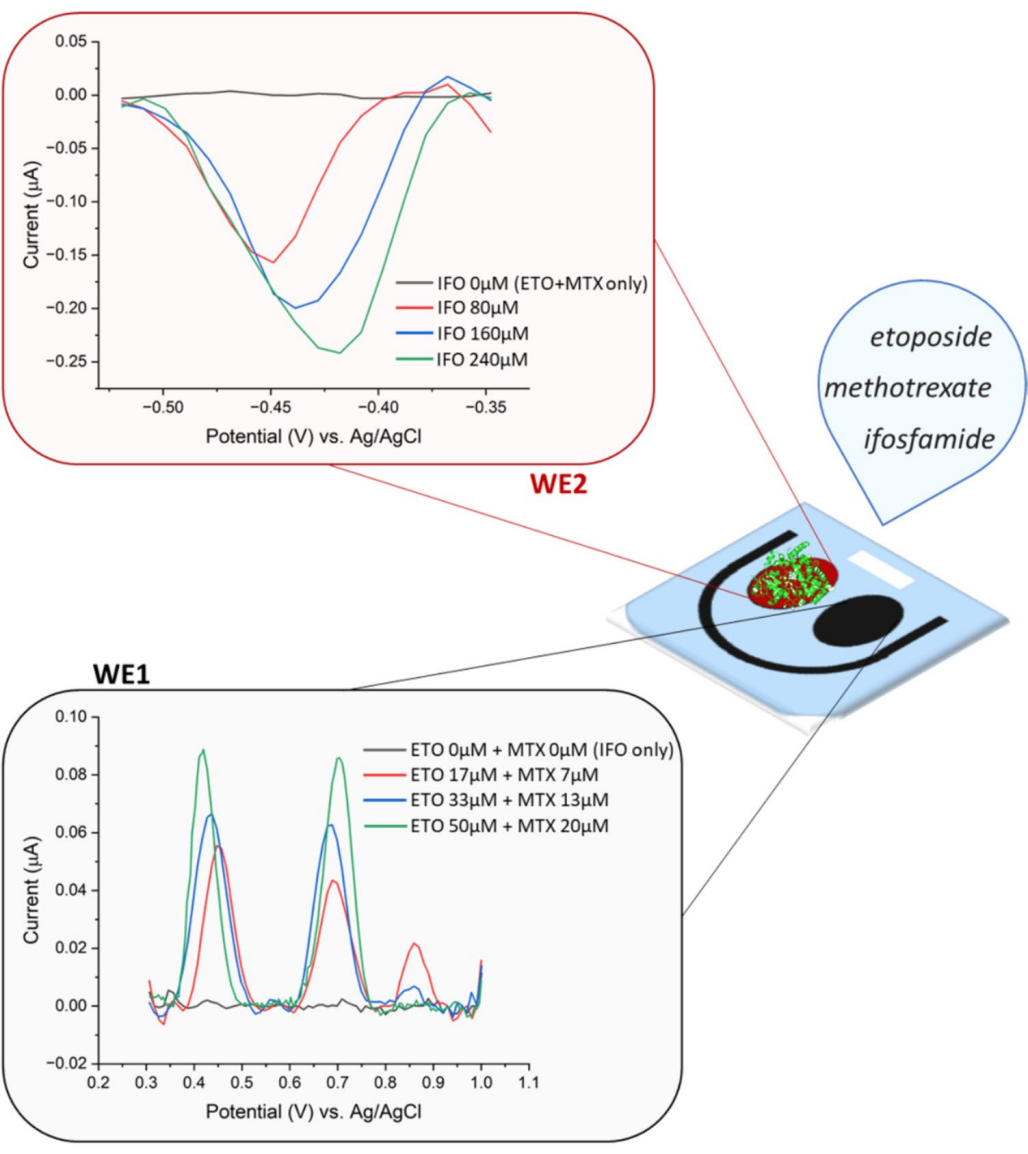
Dual-biosensor measurements for simultaneous detection of ETO, MTX, and IFO

To experimentally validate this architecture, the proposed dual-biosensor was tested using ETO, MTX, and IFO as model analytes for monitoring, reflecting their use in real chemotherapy protocols (e.g., MIED and ICE in Table II). The results in Fig. 6 validate the dual-biosensor concept by demonstrating selective and simultaneous detection of ETO, MTX, and IFO within a single measurement. In the figure, the non-enzymatic channel (WE1) clearly shows the oxidation peaks of ETO and MTX, while the enzymatic channel (WE2) responds selectively to IFO through its CYP-mediated reduction signal. Moreover, as can be observed from the gray lines in Fig. 6, there is no significant interference in the potential range where the enzymatic signal of WE2 is observed when ETO (50$\mu$M) and MTX (20$\mu$M) are present. Similarly, the response in the potential range of the WE1-detected oxidation peaks remains flat in the presence of IFO (240$\mu$M) alone, confirming that the presence of other drugs does not influence the signal on either channel. This absence of cross-talk confirms that enzymatic and non-enzymatic processes can operate independently on the same detection platform, allowing accurate quantification of each compound in mixed-drug samples. This dual-channel approach not only reduces the number of sensors required for the analysis of multiple drugs, but also minimizes sample handling and analysis time, making it highly attractive for point-of-care TDM in complex chemotherapy regimens.

## Conclusions

By tailoring therapies to the characteristics of individual patients, precision oncology aims to optimize the personalization of drug selection, dosing, and therapeutic combinations. For this purpose, TDM of administered drugs is essential to guide and adjust treatment effectively. In this work, we presented an original approach based on a novel dual-biosensor platform designed for the simultaneous detection of multiple chemotherapeutic agents for precision oncology following real clinical scenarios of oncological treatments. Unlike traditional approaches that require multiple sensors or complex configurations, our design integrates MWCNTs and CYP enzymes to achieve highly selective and sensitive detection of five widely used anticancer drugs: cyclophosphamide, ifosfamide, etoposide, methotrexate, and 5-fluorouracil. In particular, we showed both individual and combined detection, achieving excellent linearity between concentration and current response for each drug, with detection limits suitable for TDM in oncology. In addition, the ability to monitor complex drug cocktails, such as the combination of etoposide, methotrexate, and 5-fluorouracil on a single sensor, represents a significant step toward personalized and adaptive cancer care in complex chemotherapy scenarios. The presented approach represents a promising advancement in the field of precision oncology, although certain aspects merit being mentioned for further investigation. While this method has been tested in PBS samples with known analyte concentrations, it is important to consider the potential impact of complex biological matrices, such as blood or plasma, on sensor performance. Future work will then focus on validation with patient samples to address the complexities of real biological matrices. Overall, this work lays the foundation for a new class of compact, cost-effective, and highly versatile biosensors tailored for real-time TDM at the point-of-care. In oncology, where the use of multidrug regimens is standard practice, the ability to monitor several chemotherapeutic agents simultaneously using a simplified dual-electrode system represents a significant advancement. This design not only reduces the complexity and cost of traditional monitoring systems but also enhances their potential for integration into point-of-care devices for the management of personalized chemotherapy in precision oncology.

## Data Availability

No datasets were generated or analysed during the current study.

## Declarations

### Research Involving Humans and Animals Statement

This study did not involve human participants or animals.
